# Osteoid osteoma of the lunatum mimicking Kienböck’s disease

**DOI:** 10.3109/23320885.2014.993647

**Published:** 2015-01-06

**Authors:** Mehmet Derviş Güner, Haldun Onuralp Kamburoğlu, Umut Bektaş, Şadan Ay

**Affiliations:** ^a^ Department of Orthopaedics and Traumatology, Medicana International Hospital, Ankara, Turkey; ^b^ Department of Plastic Reconstructive and Aesthetic Surgery, Hacettepe University Faculty of Medicine, Ankara, Turkey

**Keywords:** Hand, tumor, osteoid osteoma, wrist

## Abstract

Hands, especially lunatum, are involved very rarely with osteoid osteoma. This report presents an osteoid osteoma of the lunatum, which was previously misdiagnosed as Kienböck’s disease and had undergone surgery. Magnetic resonance imaging may lead the clinician to misdiagnose because of the excessive bone edema around the carpus. The operation should be planned according to radiography and computed tomography findings.

## Introduction

Osteoid osteoma is a well-known benign bone tumor. It is the third most common tumor, with a percentage of 12% among all benign tumors. It has 2:1 male-to-female ratio [1]. Hands, especially lunatum, are involved very rarely. The diagnosis of osteoid osteoma in the carpus can be difficult. Patients are often misdiagnosed to have tenosynovitis or arthritis and would be mistreated [2, 3]. This report presents an osteoid osteoma of the lunatum, which was previously misdiagnosed as Kienböck’s disease and the patient had undergone radial shortening with vascularized bone graft surgery.

## Case report

A 46-year-old plasterer was referred to our clinic with a history of left wrist pain for a year. The patient stated that his complaints have occurred after hard work. There was no history of major trauma. Previously he was misdiagnosed as having Kienböck’s disease. Six month ago he had a radial shortening procedure and vascularized bone graft (implanted in lunatum) surgery for the management of this misdiagnosis in another clinic. Vascularized bone graft had been harvested from dorsal side of the radius as in Zaidemberg’s procedure [4].

**Figure 1.**
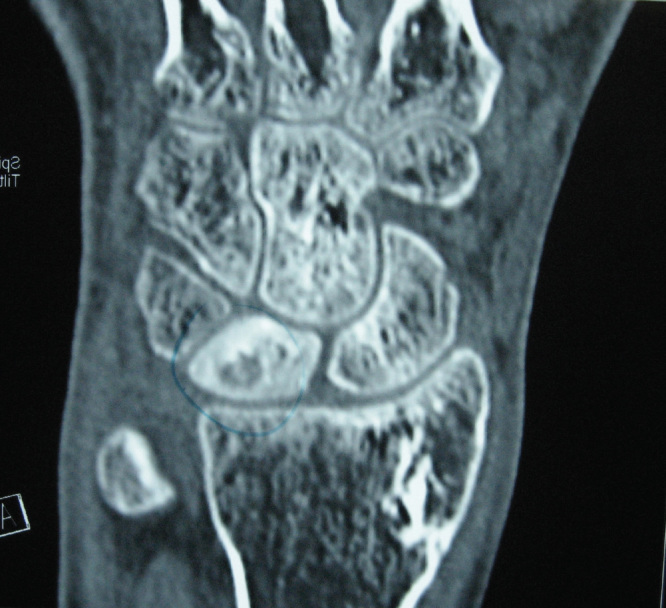
**Magnetic resonance imaging view showing the patient’s wrist before first operation. Misdiagnosis had occurred due to this view in another hospital.**

**Figure 2.**
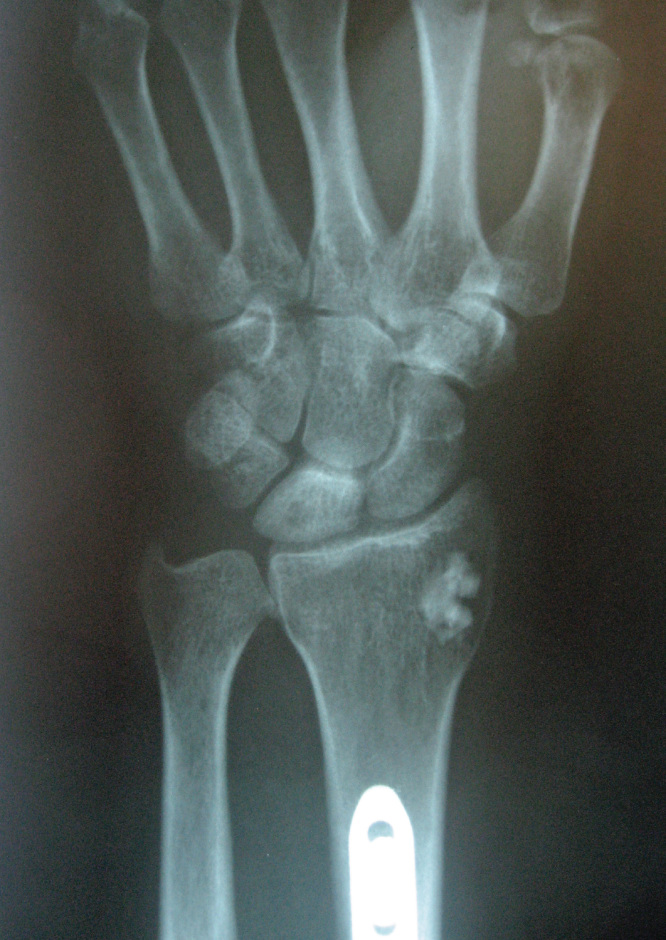
**X-ray showing the patient’s wrist after the first operation. Radial shortening and also the donor site of the bone flap can be seen.**

During physical examination, tenderness was found on the dorsal side of the wrist. Wrist dorsiflexion was limited and painful. The patient’s previous X-ray studies were normal but magnetic resonance imaging (MRI) showed severe bony edema at lunatum (Figure 1). Donor site of vascularized bone graft at distal radius and plate-screws at radius diaphysis were clearly seen in his previous postoperative X-rays (Figure 2).

**Figure 3.**
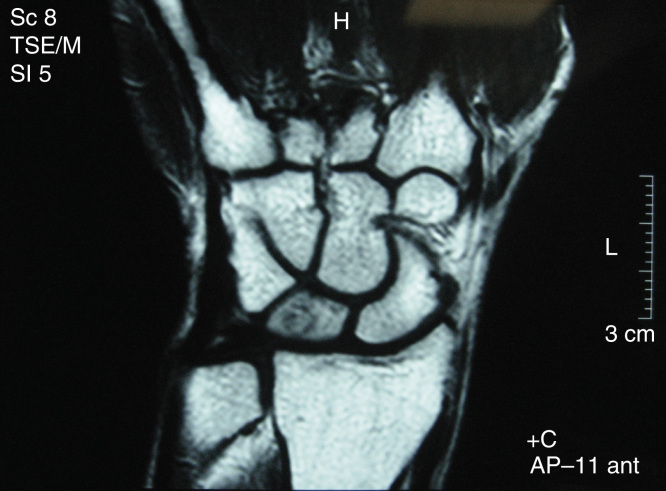
**Preoperative computed tomography scan showing the patient’s wrist before second surgery. Nidus of the osteoma can easily be seen.**

**Figure 4.**
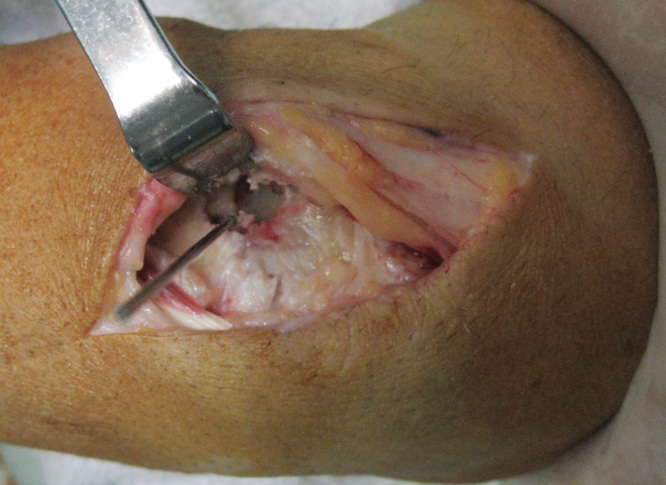
**Intraoperative view showing the tumor and nidus.**

After his referral to our clinic, computed tomography (CT) and triple-phase bone scan were performed. CT showed the nidus, as a demarcated lucent lesion, with sclerosis around it (Figure 3). It was found that previous vascularized bone graft surgery was not capable of resecting the nidus. Triple-phase bone scan revealed increased uptake at the left wrist, in all three phases. Pain subsided after acetylsalicylic acid was administered. A curettage procedure was performed in order to resect the nidus through prior incision, which was on the dorsolateral surface of the wrist (Figure 4). Histopathological examination confirmed our clinical diagnosis. Two days after the operation, pain completely subsided. There was no sign of recurrence in his 2-year follow up. Principles outlined in the Declaration of Helsinki were followed in this study.

## Discussion

Osteoid osteoma commonly affects long bones of skeleton. Hand involvement is not common. The scaphoid is the most frequently affected bone among the carpal bones [5]. Lunatum involvement is very rare [6, 7, 8, 9, 10]. On the other hand, to our knowledge, there is no report of an osteoid osteoma in lunatum which mimics Kienböck’s disease. Patients with carpal bone osteoid osteoma usually present with nonspecific wrist pain. Typically, their symptoms are worse during night and pain relief with nonsteroidal anti-inflammatory medications can be obtained. Usually, there was no remarkable medical history. Symptoms may mimic tenosynovitis or arthritis. Initial X-ray examinations are usually normal. CT is the gold standard for diagnosis and follow up [11]. Furthermore, MRI may lead the clinician to misdiagnose because of the excessive bone edema around the carpus like in our case. Although MRI is the most sensitive study, CT scan is the most specific one for hand osteoid osteoma.

The operation should be planned according to radiography and CT findings [5]. It is important to remove the entire nidus for a good clinical result. Incomplete removal of tumor may lead to persistence of pain or recurrence. Filling the defect with bone graft is unnecessary. Percutaneous radiofrequency ablation is an effective and safe minimally invasive treatment option for osteoid osteoma [12]. Recently, this CT-guided procedure has become the standard treatment for many anatomical sites [13]. High clinical success rates can be achieved with this minimally invasive and cost-effective technique [14].
